# Multicentric experience with interferon gamma therapy in sepsis induced immunosuppression. A case series

**DOI:** 10.1186/s12879-019-4526-x

**Published:** 2019-11-05

**Authors:** Didier Payen, Valerie Faivre, Jordi Miatello, Jenneke Leentjens, Caren Brumpt, Pierre Tissières, Claire Dupuis, Peter Pickkers, Anne Claire Lukaszewicz

**Affiliations:** 1 Groupe Hospitalier Saint-Louis Lariboisière, AP-HP, Université Paris 7 Denis Diderot, 2 rue Ambroise Paré, 75010 Paris, France; 2 UMR INSERM 1160 University Paris 7 Denis Diderot, Paris, France; 3 Pediatric Intensive Care and Neonatal Medicine, Bicêtre Hospital, AP-HP, Le Kremlin-Bicêtre, France; 4 Institute of Integrative Biology of Cell, CNRS, CEA, Univ. Paris Sud, Paris Saclay University, Gif sur Yvette, France; 5 Departments of intensive care and internal medicine, Radboud university medical center Nijmegen, PO box 9101, 6500 HB Nijmegen, The Netherlands; 6 Service d’Hématologie Biologique, Pôle B2P, Hôpital Lariboisière, APHP, Paris, France; 7 Department Intensive Care Medicine, Radboud university medical center Nijmegen, PO box 9101, 6500 HB Nijmegen, The Netherlands

**Keywords:** Interferon gamma, Immuno-depression, MHC class II, Cytokines, Lymphocyte immuno-phenotyping, Sepsis

## Abstract

**Background:**

The sepsis-induced immunodepression contributes to impaired clinical outcomes of various stress conditions. This syndrome is well documented and characterized by attenuated function of innate and adaptive immune cells. Several pharmacological interventions aimed to restore the immune response are emerging of which interferon-gamma (IFNγ) is one. It is of paramount relevance to obtain clinical information on optimal timing of the IFNγ-treatment, −tolerance, −effectiveness and outcome before performing a RCT. We describe the effects of IFNγ in a cohort of 18 adult and 2 pediatric sepsis patients.

**Methods:**

In this open-label prospective multi-center case-series, IFNγ treatment was initiated in patients selected on clinical and immunological criteria early (< 4 days) or late (> 7 days) following the onset of sepsis. The data collected in 18 adults and 2 liver transplanted pediatric patients were: clinical scores, monocyte expression of HLA-DR (flow cytometry), lymphocyte immune-phenotyping (flow cytometry), IL-6 and IL-10 plasma levels (ELISA), bacterial cultures, disease severity, and mortality.

**Results:**

In 15 out of 18 patients IFNγ treatment was associated with an increase of median HLA-DR expression from 2666 [IQ 1547; 4991] to 12,451 [IQ 4166; 19,707], while the absolute number of lymphocyte subpopulations were not affected, except for the decrease number of NK cells 94.5 [23; 136] to 32.5 [13; 90.8] (0.0625)]. Plasma levels of IL-6 464 [201–770] to 108 (89–140) ng/mL (*p* = 0.04) and IL-10 from IL-10 from 29 [12–59] to 9 [1–15] pg/mL decreased significantly. Three patients who received IFNγ early after ICU admission (<4 days) died. The other patients had a rapid clinical improvement assessed by the SOFA score and bacterial cultures that were repeatedly positive became negative. The 2 pediatric cases improved rapidly, but 1 died for hemorrhagic complication.

**Conclusion:**

Guided by clinical and immunological monitoring, adjunctive immunotherapy with IFNγ appears well-tolerated in our cases and improves immune host defense in sepsis induced immuno suppression. Randomized clinical studies to assess its potential clinical benefit are warranted.

## Background

One of the major advances in the field of sepsis during the last decade is the accumulation of evidence demonstrating the early shift in immune phenotype from an activated to a suppressed phenotype soon after the onset of sepsis [1]. Although the molecular mechanisms are not fully elucidated yet, the reported immune dysfunction of the cells from septic patients during immune-depression concern both the innate and adaptive immunity [2]. This phenotype is also observed in many other acute injuries suggesting a common pathophysiology after significant stress conditions as trauma [3], cardiopulmonary resuscitation [4], and major surgery [5]. However, if immunodepression is profound and persistent the patients become at risk of secondary infections [6] or are unable to resolve the primary infection [7].

To assess the patients’ immune status, immune monitoring becomes essential. Until now, there are few adequate “immunoscope” candidates that are feasible in daily clinical practice and may facilitate the decision-making process. The quantification of the expression of the MHC class II molecule HLA-DR on circulating monocytes appears to represent a suitable parameter [8–11]. A persistent low level of HLA-DR expression is associated with the development of secondary infections and impaired clinical outcome [6, 12]. Of importance, it is becoming increasingly clear that the attenuated immune response can be reversed by different pharmacological interventions, including GM-CSF [13], IL-7 [14], anti-PD [15] and IFNγ [16]. Nowadays, as a last resort treatment, immunostimulating treatment is sometimes applied in critically ill sepsis patients and several small case series have been published [16]. Although effects on clinically relevant end-points can only be determined in randomized clinical trials, relevant data related to immunological properties, safety, and timing of treatment are needed to facilitate the design of such trials.

As previously reported [9], we choose the recombinant human IFNγ to treat immunodepression in different cases phenotype from 3 tertiary hospitals, including children in a context of liver transplantation. This molecule was selected for several reasons: 1- it is already therapeutically used for immune deficiency [17]; 2- the previous case reports did not mention severe side effects; and 3- the impact on innate immunity could be well monitored by the monocyte expression of HLA-DR. [10]

## Methods

### Patients and methods

Two different cohorts of septic patients (sepsis 2 definition) were collected. The *Cohort 1* of 13 adult patients having a sepsis-induced immunodepression syndrome was collected from May 2004 till 2017 in the Surgical Intensive Care (SICU) at Lariboisière University Hospital, Paris, France from the large project “Severe Sepsis and inflammation monitoring” approved by Cochin Hospital Ethics Committee (# CCPPRB 2061), Assistance Publique Hôpitaux de Paris. The decision to administer IFNγ was made on the following criteria, which were not modified between 2004 and 2017: (1) an ICU stay over 7 days; (2) a diagnosis of secondary infection/colonization or an uncontrolled initial infection despite adequate antimicrobial therapy and/or interventional procedures; (3) a stable (at least 2 measurements) low level of mHLA-DR expression (<8000 antibody bound/cell (AB/C in our laboratory). Before IFNγ treatment (100mcg per subcutaneous injection, repeated at least 3 days with a maximum duration of 5 days) a written informed consent was obtained for each individual or from closest relative. The clear explanation of the potential risks and benefits to administer the drug as a compassionate treatment was applied according to the French Ethical law. For some patients, pro- and anti-inflammatory plasma cytokines levels were measured before and just after the end of IFNγ treatment. For the first time, the impact on lymphocyte immune phenotype was also evaluated.

The second *Cohort 2* had 4 patients from the Radboud University Medical Centre (Nijmegen, Netherlands). The patients were hospitalized for septic shock (Sepsis 2 definition) and were enrolled in a randomized clinical pilot trial (NCT 01649921). When norepinephrine infusion rate was reduced to 50% of maximum dose, ensuring that the sepsis-induced inflammation was declining (day 0), the administration of IFNγ (100mcg subcutaneous/day) was started. As a consequence, patients in this cohort could be treated with IFNγ earlier the day 7 in the ICU. This pilot trial was prematurely terminated due to a low enrollment rate.

In addition, 2 pediatric cases from the Pediatric Intensive Care (Kremlin-Bicêtre University Hospital) were added. Case 1: a 7 y/o transplanted the 1st time at the first year for fulminant hepatitis had to be transplanted again for chronic humoral rejection despite full treatment. She was referred for end-stage liver failure motivating an emergency liver transplantation 1 month after admission outlined by a hemorrhagic shock. After transplantation, continuous veno-venous hemodiafiltration was used for anuric renal failure and massive fluid overload. Under post-operative immunosuppression (basiliximab on day 1 and 4, methylprednisolone, tacrolimus and mycophenolate mofetil) an invasive aspergillosis (*Aspergillus fumigatus*) was confirmed in BAL, lumbar puncture and blood PCR with serial galactomannan antigenemia and blood PCR. Antifungal therapy associating voriconazole and caspofungine were initiated and immunosuppression stopped. The child developed an acute and reversible hemorrhagic event related to multifocal intracerebral Aspergillosis lesions, and severe ARDS. In regard to the severity of the infection, rescue IFNγ was considered and proposed as a compassionate treatment. After parent’s agreement and despite the modest reduction in mHLA-DR expression (mHLA-DR: 15,000 AB/C), one dose of 100 micrograms of IFNγ was subcutaneously injected. Although the improvement of the child condition (resolution of the respiratory failure and clinical awakening allowing to extubate), the child died on day 18 after transplantation with uncontrolled hemorrhage. Case 2: a 22-month-old boy originally transplanted for biliary atresia, was admitted to be transplanted again for recurrent cholangitis secondary to post-transplantation hepatic artery thrombosis and ischemic cholangitis.. The postoperative complications were: a renal tubulopathy; a delayed awaking and a suspicion of ventilatory associated pneumonia. He was rapidly re-intubated because of developed ARDS, associated with anuria, and septic shock with severe hypoxemia despite the use of all techniques to improve oxygenation. Distal lung protected sampling (> 10^7^ cfu/ml) and ascites were positive for highly resistant *Pseudomonas aeruginosa*. Maximal supportive therapy associated with iv and aerosolized colistin with potential acquisition of resistance motivated discussion about IFNγ treatment. Monocytic HLA-DR measures were repetitively low (Day 11: mHLA-DR 2773 AB/C) (Fig. 2), 20 micrograms of IFNγ were injected subcutaneously during three consecutive days, with no significant side effect. The child dramatically improved hemodynamically at day 1 after the first IFNγ injection and was extubated 2 days later. Repetitive blood cultures and lung and abdominal samples remained negative. The child remains well thereafter. Both patients’ immune monitoring of HLA-DR was monitored by the center A with the same protocol than cohort 1, using the same set up of flowcytometer.

### IFNγ treatment

According to previous publication in human sepsis, a dose of 100mcg of IFNγ (Imukin®, Boehringer, Ingelheim, Germany) was subcutaneously injected for 3 to 5 days (cohort 1) or on days 2–4–7-9-11 (cohort 2) as reported [6, 9, 18, 19]. The treatment was repeated to reach an increased mHLA-DR expression above 8000 AB/C (cohort 1) or MFI > 20 in cohort 2 (low threshold fixed at MFI 20). Clinical tolerance and systemic or local (injection site) symptoms of inflammation were carefully checked.

### Immune monitoring

The mHLA-DR expression was measured by flow cytometry (FACSCalibur and FACSCanto, Becton Dickinson, San Diego CA) as previously described in detail [6]. For the Lariboisière center (cohort 1), the median and IQ range of mHLA-DR expression in healthy people (*n* = 50) was 16,884 [13,255–20,890] antibody bound per cell (AB/C). In cohort 2, mHLA-DR expression was assessed by the Mean Fluorescence Index (MFI, flow cytometry).

Plasma levels of IL-6 and IL-10 (ELISA method) were measured before, during and the day after the IFNγ treatment cessation in 6 patients of the cohort 1. The impact of IFNγ on lymphocyte subpopulations (CD3, CD4, CD8, CD19, and NK) was also investigated in 6 patients using classic immune-phenotyping method (see Additional files 1 and 2). The results were expressed in absolute values of subpopulations (central laboratory immune-phenotyping).

Because of the relatively small sample size, normal distribution could not be assumed and data is reported as median and interquartile range [IQR]. The non-parametric tests (Mann Whitney and Wilcoxon tests) were used appropriately to check the significant changes over time. Statistical SAS software, version 9.3 (SAS Institute Inc., Cary, North Carolina, USA) was used.

## Results

**Fig. 1:**
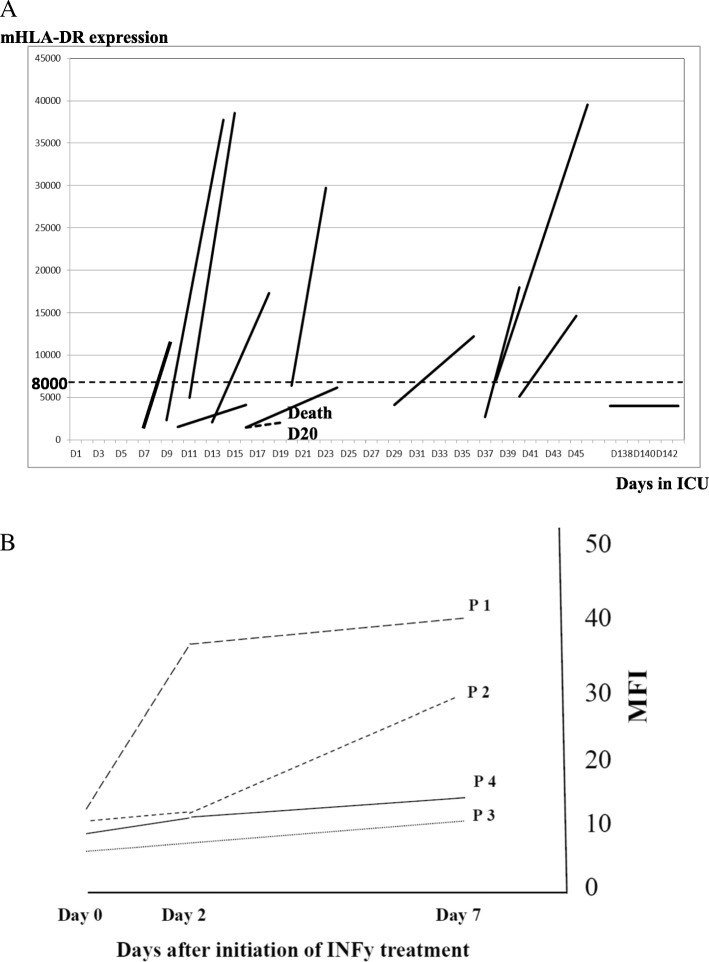
shows the evolution of monocyte HLA-DR expression. Figure 1**a** (cohort A) depicts the individual data of mHLA-DR expression (AB/C event/cell) before and within the 24 h after stopping IFNγ treatment, showing the real delay from the admission to be treated. The dotted line figures the threshold below which the immunodepression is identified. Among the 13 patients, 4 increased the HLA-DR expression but did not reach the defined threshold. The X axis: days from admission; the Y axis represents the quantitative AB/C values of mHLA-DR expression. Figure 1**b** (cohort B) shows similar representation: the X axis: days were IFNγ treatment was administered; Y axis represents the MFI level

Table 1 shows the characteristics of the cohort 1 and 2 of adult patients. In cohort 1, beside a slight increase in baseline body temperature (pre-treatment value 37.8 °C (37.5–38.4), the temperature increased to 39 °C (38.1–39.7), *p* < 0.05) during IFNγ treatment, no other side effects were observed. Figure 1a shows the individual mHLA-DR expression of the cohort 1 before and the day after stopping IFNγ stimulation with the delay from the ICU admission day. No relation between the time delay or the baseline level of mHLA-DR expression was observed for the drug response. All except one patient with a previously diagnosed Waldenström disease, increased their mHLA-DR expression. This non-responder patient to IFNγ died 3 days after the treatment initiation. Nine out of 13 survivors increased their mHLA-DR above the threshold of 8000 AB/C. In the remaining 4 patients, the HLA-DR expression increased, but not sufficiently to overpass the lower threshold limit and the treatment was stopped. Nevertheless, microbial cultures that remained positive prior to IFNγ treatment became negative in all patients. The bacterial cultures performed 6 days after the onset of IFNγ treatment (*i.e* 1 to 4 days after treatment) were all negative. The daily collected SOFA scores before during and after IFNγ administration decreased in 10 of 13 patients (Additional file 3: Figure S1). Only one patient having responded adequately to IFNγ treatment developed 6 days later a new pulmonary infection with positive cultures (*Pseudomonas aeruginosa*, Klebsiella and Aspergillus fumigatus) and recurrent immunodepression. This new infection episode occurred despite maintenance of adequate antimicrobial treatment concomitantly with a drop in mHLA-DR expression (Additional file 4: Figure S2). The medical team decided to restart the IFNγ treatment for 4 days inducing again the resolved infection accompanied by a quick improvement in clinic and in chest CT scan (Additional file 4: Figure S2). After discharge from ICU, 4 patients died at day 52, 86, 108 and day 176 post admission.

**Table 1:** Clinical and infection characteristics of patients treated with IFNγ for cohort 1 and Cohort 2. AB/C = antibodies per cell. MFI = Mean Fluorescence Intensity

Cohort 1									
	Age	Diagnosis at admission	Day of ICU	Secondary infection	microorganism	Antibiotic treatment	mHLA-DR (AB/C)	Injections of IFNγ	Day-15 outcome
1	30	Cardiac arrest	10	*VAP*	*Pseudomonas aeruginosa*	amikacin (4 days) + colimycin (2 days) + cefepim (2 days)	2419	5	alive
2	83	Postop cardiogenic shock	29	*VAP*	*Pseudomonas aeruginosa, Stenotrophomonas maltophilia*	ciprofloxacin (11 days) + ceftazidime (13 days)	4092	6	alive
3	73	Cardiogenic shock	16	*VAP*	*Stenotrophomonas maltophilia, Pseudomonas aeruginosa*	piperacillin + tazobactam (4 days)	1492	4	alive
4	63	Peritonitis	16	*VAP*	*Pseudomonas aeruginosa*	Imipenem (7 days)	1427	3	dead
5	42	Peritonitis	10	*VAP, peritonitis*	*Pseudomonas aeruginosa, Aspergillus fumigatus*	piperacillin+ tazobactam (10 days)	1547	6	alive
6	64	Peritonitis	37	*Peritonitis* *VAP*	*Stenotrophomonas maltophilia + Enterobacter Cloacae + Enterococcus feacalis* *Enterobacter cloacae + Stenotrophomonas maltophilia*	tigecycline + colimycin	2666	3	alive
7	65	Postoparative pneumonia	134	*VAP*	*Streptococcus agalactiae*	none	3289	5	alive
8	56	Pneumonia	11	*VAP*	*Stenotrophomonas maltophilia* *EBV reactivation*	piperacillin + tazobactam (10 days)	4991	4	alive
9	34	Pneumonia	15	*VAP*	*Pseudomonas aeruginosa* *Aspergillus fumigatus*	colimycine (15 days) amikacine (aerosolized) 15 days Voriconazole started	5428	5	alive
10	56	Cervical cellulitis septicemia	13	*Perirenal abscess*	*Staphylococcus aureus*	oxacilline + Pefloxacine (12 days)	2056	7	alive
11	60	Fasciitis	38	*Fasciitis*	*Pseudomonas aeruginosa*	Imipenem + amikacine (3 days)	5132	5	alive
12	74	Keto-acidosis	40	*VAP, lung abcess, pleuresis*	*Pseudomonas aeruginosa, Citrobacter freundii*	Imipenem (7 days)	7073	4	Alive
13	82	Rectal Fistulae & fasciitis	9	*Tight muscle infection*	*Gram negative multiple bacteriaanaerobes*	Piperacillin + tazobactam (4 days) Imipenem + amikacine (7 days)	2168	3	Alive
Cohort 2									
Age		Diagnosis at admission	Infection focus	Delay between admission and IFNγ	Microorganism	Antibiotic treatment	SOFA-score at admission	mHLA-DR Expression (MFI)	Outcome
74		Septic Shock	Abdominal	4	Unknown	Ceftriaxon	12	16.0	Death
73		“	Abdominal	3	*Klebsiella pneumoniae*	Ceftriaxon	8	14.6	Alive
74		“	Bilary	1	Multi-resistant *E coli, Clostridium perfringens*	Piperacillin / tazobactam, ceftriaxon erytromycin	14	5.6	Alive
80		“	Abdominal	5	*Staphylococcus haemolyticus, Candida albicans*	Vancomycin and Piperacillin / tazobactam, myfungin / fluconazole	9	73.6	Death

*VAP* Ventilator-associated pneumonia, *EBV* Ebstein Barr virus, *CMV* cytomegalovirus

**Fig. 2:**
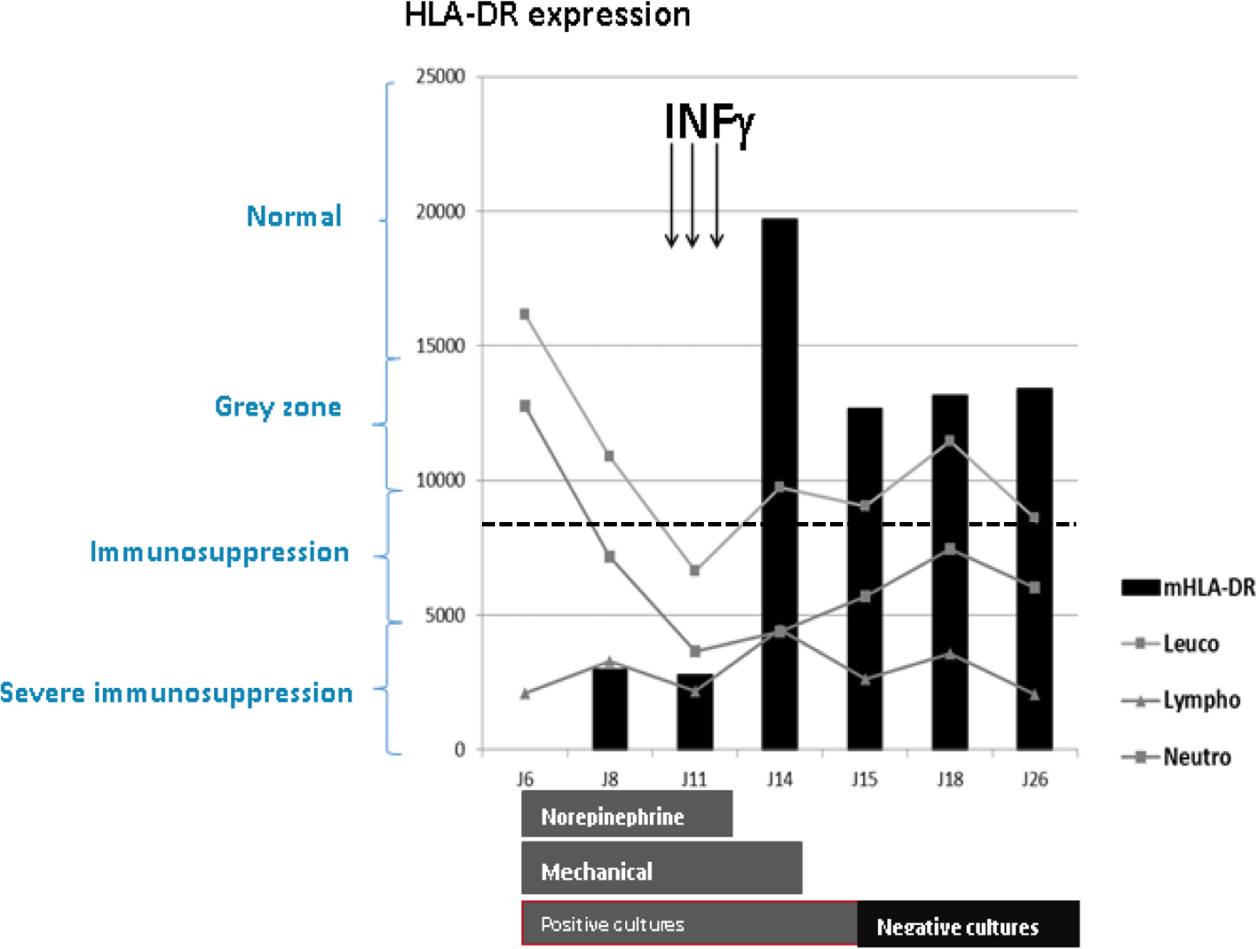
Schematic representation of the evolution of the pediatric case 2 before and after IFNγ treatment. The dotted line depicts the threshold used to define a significant immunodepression

In cohort 2, in accordance with the protocol (NCT 01649921) the 1st dose of IFNγ was administered between day 1 and 4 after admission to ICU for septic shock. Table 1 summarizes the baseline characteristics and outcomes. The 2 cohorts were different for several items: the cohort 2 was older with a higher APACHE II score level and dose of norepinephrine infusion at time of decision to use IFNγ. Figure 2b depicts the individual evolution of mHLA-DR levels expressed in MFI of variations from pre-treatment values. Initial mHLA-DR levels were low (below MFI 20) in 3 out of 4 patients and increased during IFNγ treatment. In one patient the IFNγ-induced a rapid increase in mHLA-DR expression, promptly followed by a sharp decrease of HLA-DR expression, associated with clinical deterioration and death. Two patients died within the 14-days after initiation of IFNγ treatment. No serious adverse events linked to IFNγ treatment were observed.

### Pediatric cases

Both had uncontrolled infection after liver transplantation despite the full supportive and adequate microbial therapies. The expression of mHLA-DR demonstrated a sharp increase when IFNγ was administered. The usual immunosuppressive drugs to prevent graft rejection after liver transplantation were maintained. Figure 2 illustrates the evolution of mHLA-DR expression in the case n°2 before, during, and after administration of 20 mcg/subcutaneous injection of IFNγ for 5 days.

### Subgroup for immunological evaluation

**Fig. 3:**
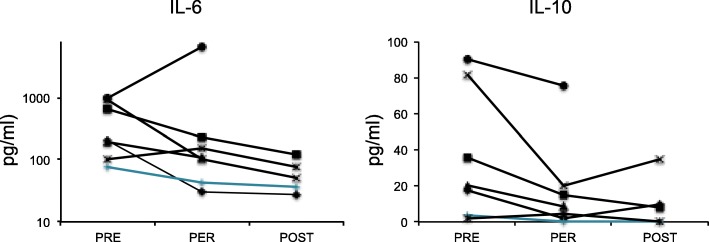
shows the individual evolution of plasma IL-6 and IL-10 before (PRE) during (PER) and 24 h after (POST) IFNγ treatment of the 6 patients from cohort 1 having immunophenotyping

**Fig. 4:**
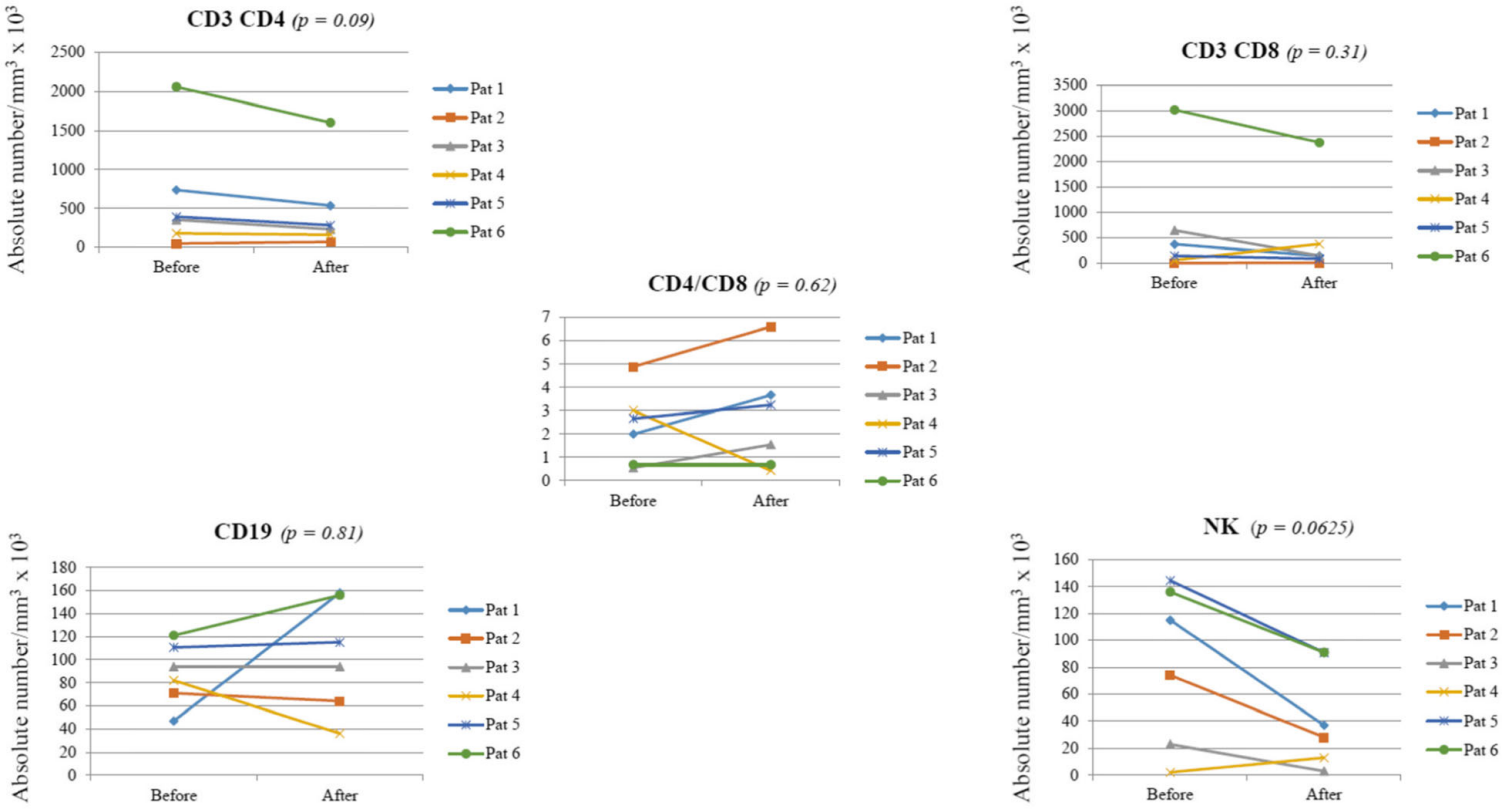
depict the individual immunophenotyping data (cohort 1; *n* = 6) before and 24 h after IFNγ expressed in absolute number of cells in Y axis or ratio for CD4/CD8. CD3: Lymphocyte; CD4: Lymphocyte T4; CD8: Lymphocyte T8; CD 19: Lymphocyte B; NK: Lymphocyte Natural Killer assessed by flowcytometry

**Fig. 5:**
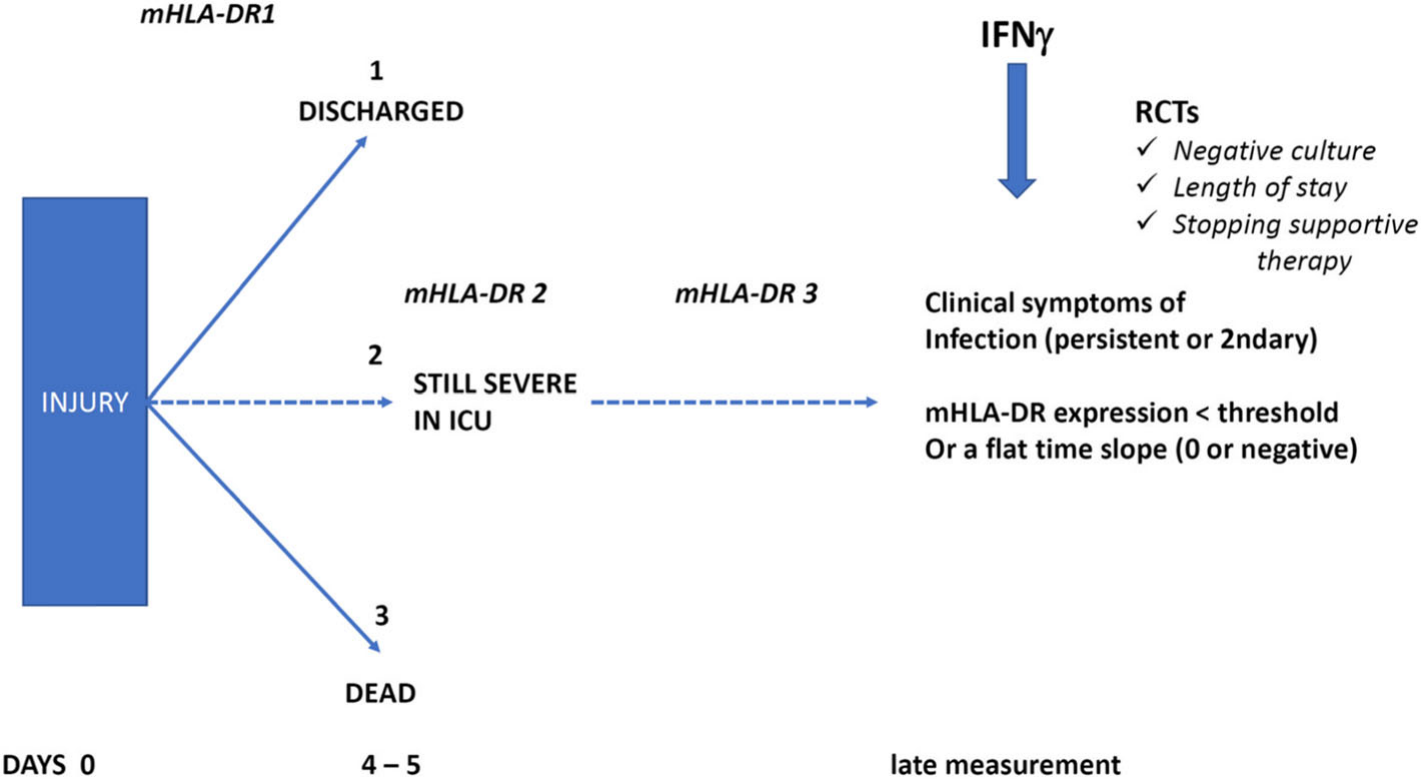
schematic representation of the targeted population potentially selected to perform randomized clinical trials (RCTs). The baseline mHLA-DR expression has to be measured during the first 4–5 days post-admission. Patients remaining in intensive care (ICU) after 5 days have sequential mHLA-DR measurements to evaluate the trend of evolution. Both a low mHLA-DR level or a flat/negative trend associated with symptoms of infection despite adequate treatment, leads to consider the use of IFNγ and could be randomized

Figure 3 summarizes the course of individual plasma IL-6 and IL-10 levels from baseline to the day post IFNγ treatment (*n* = 6 from cohort 1). IL-6 decreased from 464 [201–770] to 108 (89–140) ng/mL (*p* = 0.04) with a decrease in IL-10 from 29 [12–59] to 9 [1-15] pg/mL (*p* = 0.06). Figure 4 shows the individual evolution of the patients from the cohort 1 having lymphocyte immune-phenotyping. Treatment with IFNγ did not modify the %, nor the absolute value of lymphocyte subpopulations, except for NK cells. IFNγ treatment induced a clear trend of decreased absolute value of NK cell subtype from 13 [12-14] to 10 [3-10] (*p* = 0.0625). Figure 5 is a schematic proposal to consider a potential treatment for acute severe immunodepression that could be applied for future randomized control trials.

## Discussion

Over the last decades it is increasingly recognized that a large proportion of sepsis patients suffer from a sustained suppression of their immune system [1]. This immune-suppression is associated with reactivation of latent viral infections [20], the development of secondary infections and impaired clinical outcomes [6, 21]. Several pharmacological interventions may restore the immune response, but adequately large clinical trials are currently lacking. We investigated the safety and immunological effects of IFNγ in 17 adult patients reported that IFNγ increased monocytic HLA-DR expression in all except one patient. Treatment with IFNγ did not boost circulating cytokine concentrations: IL-6 and IL-10 concentrations were lower than pretreatment values. It tended to decrease NK-cell proportion without changes for other leukocyte subpopulations. In addition, cultures that remained positive prior to IFNγ treatment became negative in all patients. No safety effects judged to be attributable to IFNγ were observed. Overall, survival appeared higher than anticipated in this specific group of immunoparalyzed patients, but clearly the numbers are too small to conclude that the restoration of the immune response is related to better clinical outcome.

The IFNγ used in the present case series was a human type II IFNγ having the advantage to be 100–1000 more active than other interferons [22]. This molecule received the FDA approval for clinical application against chronic granulomatous disease to decrease the severity and number of infections. Beneficial effects of adjuvant treatment using IFNγ for fungal infections in patients suffering from chronic granulomatous disease [17] HIV [23–25], leukemia [26, 27], and in organ transplant patients [28] were previously reported. The first report using this IFNγ in human bacterial sepsis was made by Docke et al. [9] in 9 septic ICU patients at 100mcg daily dose. Since then, several small case series suffering from opportunistic infections [16] [29] or pediatric case [30, 31] or HIV cases [23–25] have been published. The present case series is the first reporting the use of IFNγ in adult and pediatric bacterial sepsis, including septic shock patients with associated cytokines level modifications and immunophenotyping. The delay to use IFNγ from the ICU hospitalization differed largely within the 2 reported small cohorts. One might argue that the unfavorable outcome in 2 patients of cohort 2 early treated may suggest that IFNγ therapy should not be used too early, even when HLA-DR expression is already suppressed. In the early treated group, the patients are still in the steep part of the survival curve with a high mortality (close to 50%) of severe septic shock patients [32]. Nevertheless, the cases of the cohort 2 early treated (< 4 days after admission for septic shock) had a rapid downregulation of HLA-DR expression that responded well to IFNγ stimulation for mHLA-DR expression. The lack of positive signal for outcome could be related to a downregulation adapted to the acute phase with no benefit to administer IFNγ.

The concentrations of circulating cytokines were measured in cohort 1 to examine whether or not the immunostimulating with IFNγ would result in increases in these cytokines, which could have deleterious effects. This was not the case, both circulating IL-6 and IL-10 levels decreased following IFNγ treatment. In accordance, in volunteers exposed to endotoxin twice, treatment with IFNγ restored the suppressed cytokine response during the second endotoxin exposure. So, IFNγ prevents and reverses the in vivo induced endotoxin tolerance with no indications of an overshoot response [18]. The modest changes induced by IFNγ in lymphocytes subpopulations were observed, except for absolute number of NK cells. The clear trend of NK cell decrease in our case series may result from a migration into infected tissue or from a reduction in NK cell generation. Being poorly reported, this observation made in a small size cohort should be cautiously interpreted. The reported case series on invasive fungi infection did not show changes in the total leucocyte and granulocyte numbers when IFNγ-treatment was given change [16].

The predominant effect on innate immunity associated with the moderate changes of adaptive immunity, opens the possibility to use IFNγ even after organ transplantation maintaining the anti-rejection treatment targeting mainly the lymphocytes. It is possible and reasonable to combine the IFNγ treatment with the maintenance of an anti-graft rejection treatment when an infection cannot be controlled.

The clinical benefit of IFNγ treatment appeared favorable in cohort 1 with only 1 death. All cohort except 1 patient improved enough to be discharged from ICU. This observation suggests that IFNγ is most clinically effective if the suppressed immune response is present for a longer period and is associated with new infections. We hypothesized that the duration more than the depth of mHLA-DR downregulation is relevant for the clinician to detect the risk of new infections. This aspect should be verified in a larger population. As reported previously, the IFNγ treatment was well tolerated in our 20 patients with no clearly attributable severe side effects. The monitoring of HLA-DR expression on circulating monocytes appears adequate to follow the immunological efficacy of the compound, a relatively cheap immune monitoring exam to facilitate the clinical decision making.

This case series has important limitations, mostly related to its observational nature and the compassionate use of the drug. First, the 20 collected cases are too limited to draw definitive conclusions about the clinical benefit of this treatment. However, grouping together the case reports and the other case series results in at least 50 reported cases treated by IFNγ. Overall, an excellent tolerance was reported using a 100 mcg daily in adult. It is still essential to determine the dose-response relationship, and the tolerance of potential repetitive treatment to complete the safety profile of the compound. Despite the small number of cases, it is remarkable that mHLA-DR significantly increased following the first injection and went down again rapidly after treatment cessation. Second, macrophage or monocyte polarization (M1 or M2) has not been tested in our cases, which hampered the understanding of re-programming mechanisms and its impact on monocyte-macrophage polarization.

## Conclusion

Sepsis induces suppression of the immune system, associated with susceptibility to secondary infections and impaired clinical outcome. We report cases for whom IFNγ treatment was well-tolerated and improved immune host defense. The increase in monocytic HLA-DR expression did not induce a storm of cytokine release nor a modification in lymphocyte immunophenotyping, except for decrease in absolute number of NK cells. Within the limits of small size cohort, the clinical benefit of IFNγ to stimulate innate immunity in presence of immunosuppression is an attractive track for the future. The Fig. 5 illustrates the potential design for future clinical trials. The primary end-point might be the resolution of infection and/or positive culture and the length of stay in intensive care.

## Supplementary information

**Additional file 1.** Supplementary methods

**Additional file 2: **Supplementary pediatric cases

**Additional file 3: Figure S1.** Variations in SOFA score before and after IFNy treatment in cohort A. Legend: Vertical axis: % of SOFA variations; horizontal axis: days before and after treatment by INFγ.

**Additional file 4: Figure S2.** Evolution of pulmonary CT scan over days in patient 8.

## Data Availability

According to the ethical medical protection in France, the case data cannot be diffused. The data can be obtained on specific request to the authors.

## Abbreviations

CD: Cluster of Differentiation
HIV: Human Immunodeficiency Virus
HLA-DR: Human Leucocyte Antigen-DR
ICU: Intensive Care Unit
IFNγ: interferon gamma
IL-10: interleukine 10
IL-6: interleukine 6
MFI: Mean Fluorescence intensity
MHC class II: Major Histocompatibility Complex II
NK: Natural Killer
PCR: Polymerase Chain Reaction
PD: Program Death
RCT: Randomized Clinical Trial
SICU: Surgical Intensive Care Unit
SOFA: Sequential Organ Failure Assessment
