# Supplementary material for: Multicentric experience with interferon gamma therapy in sepsis induced immunosuppression. A case series
Source: BMC Infect Dis. 2019 Nov 5;19:931. doi: 10.1186/s12879-019-4526-x (PMC6833157; doi:10.1186/s12879-019-4526-x)
Supplement: Supplementary file 1 — Additional file 1. Supplementary methods [file 12879_2019_4526_MOESM1_ESM.rtf]

Additional file 1
Methods:
Flowcytometry for mHLA-DR characterization:

Immunophenotyping: Sample processing: A complete blood count (CBC) with the White Blood Cells count was performed (including lymphocytes count) for all patients within 6h after collection. Flow cytometry : The evaluation of peripheral blood T, B and NK lymphocytes populations was estimated using FacsCanto™ II flow cytometer and  the BD MultiTESTTM 4-color Reagents from BD Biosciences (San Diego USA) :  CD3 FITC, CD8 PE, CD45 PerCP , CD4 APC (Cat no.342447) and CD3 FITC, CD16/CD56 PE, CD45 PerCP, CD19 APC (Cat no.342446). Whole blood samples (4 ml) were collected in EDTA vacutainers and processed within 24 h of collection. Twenty ìl of liquid antibody reagent and 50 ìl of whole blood was added to the BD TruCOUNTTM tube containing the reference beads. The tube was vortexed and incubated at ambient temperature in dark for 15 min. The RBCs were lysed using 450 ìl of 1:10 diluted lysing solution (BD FACS lysing SolutionTM) for 15 min in the dark at ambient temperature. The stained sample was analyzed using the automated FacsCantoClinical SoftwareTM by automated gating. The percent and the absolute count of each lymphocytes subpopulation was calculated automatically by the software.
