# Supplementary material for: Multicentric experience with interferon gamma therapy in sepsis induced immunosuppression. A case series
Source: BMC Infect Dis. 2019 Nov 5;19:931. doi: 10.1186/s12879-019-4526-x (PMC6833157; doi:10.1186/s12879-019-4526-x)
Supplement: Supplementary file 2 — Additional file 2: Supplementary pediatric cases [file 12879_2019_4526_MOESM2_ESM.docx]

Additional file 2: pediatric cases

Case n°1; 7y/o girl was re-transplanted liver, 1 month after PICU re-hospitalization, which was complicated by hemorrhagic shock, anuric renal failure and massive fluid overload justifying continuous hemofiltration. After diagnosing invasive aspergillosis (*Aspergillus fumigatus;* endotracheal aspirate culture+ antigenemia + positive blood PCR) despite adequate therapy (voriconazole and caspofungine), the pediatrician team decided to stop anti-rejection treatment and to measure mHLA-DR expression. mHLA-DR expression was above the lower range of normal values (mHLA-DR: 15,000 AB/C). Because of the context and the severity, 20mcg/day of IFNγ was injected for 3 days. After the 2nd injection, the clinical condition improved with a resolution of respiratory failure allowing to extubate the child. A second hemorrhagic shock occurred leading to death. The case n°2 was a 22 months old boy having a liver re-transplantation for recurrent cholangitis. Secondary infection with septic shock due to highly resistant *Pseudomonas aeruginosa* (carbapenem R; fosfomycin R; and colistin intermediate resistance) and catheter-related ESBL-producing *Klebsiella pneumonia* infection occurred. The measured low mHLA-DR expression (mHLA-DR 2773 AB/C) led to treat with 20 mcg/subcutaneous IFNγ. One day after, the clinical status and hemodynamic parameters dramatically improved leading to extubation at day 2. mHLA-DR expression increased rapidly and sustained (figure 4) with no side effects, especially for the liver rejection. Further blood, lung, and abdomen cultures of were negative despite continued post-transplantation immunosuppression therapy. The child was discharged from PICU.
