# Supplementary material for: Multicentric experience with interferon gamma therapy in sepsis induced immunosuppression. A case series
Source: BMC Infect Dis. 2019 Nov 5;19:931. doi: 10.1186/s12879-019-4526-x (PMC6833157; doi:10.1186/s12879-019-4526-x)
Supplement: Supplementary file 3 — Additional file 3: Figure S1. Variations in SOFA score before and after IFNy treatment in cohort A. Legend: Vertical axis: % of SOFA variations; horizontal axis: days before and after treatment by INFγ. [file 12879_2019_4526_MOESM3_ESM.docx]

Additional File 2: Figure S1

Suppl Fig 2: Evolution of SOFA score from admission expressed in %. 0% was the reference before starting INFγ injection. Vertical axis: % of SOFA variations; horizontal axis: days before and after treatment by INFγ. The pink rectangle figures the time for INFγ treatment.
