# Supplementary material for: Multicentric experience with interferon gamma therapy in sepsis induced immunosuppression. A case series
Source: BMC Infect Dis. 2019 Nov 5;19:931. doi: 10.1186/s12879-019-4526-x (PMC6833157; doi:10.1186/s12879-019-4526-x)
Supplement: Supplementary file 4 — Additional file 4: Figure S2. Evolution of pulmonary CT scan over days in patient 8. [file 12879_2019_4526_MOESM4_ESM.docx]

Additional file 3: Figure S2

**Figure S 2** Evolution of pulmonary CT scan over days in patient 8, admitted for pneumonia related to *Legionella pneumophilia* infection (**A**), complicated by a first VAP with *Pseudomonas* and *Aspergillus fumigatus* (day 15, **B**) treated by antibiotic and IFNγ (day 21 to 26). Recurrence of VAT with *Pseudomonas aeruginosa* and *Klebsiella pneumoniae* and rare *Aspergillus fumigatus* (day 35, **C**) led to the administration of a second treatment with IFNγ (day 39 to 42) associated with antibiotics to better control infection (day 44, **D**; day 58, **E**).
